# Emerging role of hypoxia-inducible factor-1α in inflammatory autoimmune diseases: A comprehensive review

**DOI:** 10.3389/fimmu.2022.1073971

**Published:** 2023-01-25

**Authors:** Yang-Yang Tang, Da-Cheng Wang, You-Qiang Wang, An-Fang Huang, Wang-Dong Xu

**Affiliations:** ^1^ Department of Evidence-Based Medicine, Southwest Medical University, Luzhou, Sichuan, China; ^2^ Department of Laboratory Medicine, The Affiliated Traditional Chinese Medicine Hospital of Southwest Medical University, Luzhou, Sichuan, China; ^3^ Department of Rheumatology and Immunology, Affiliated Hospital of Southwest Medical University, Luzhou, Sichuan, China

**Keywords:** immune cell, inflammation, autoimmune diseases, HIF-1α, signaling pathway

## Abstract

Hypoxia-inducible factor-1α (HIF-1α) is a primary metabolic sensor, and is expressed in different immune cells, such as macrophage, dendritic cell, neutrophil, T cell, and non-immune cells, for instance, synovial fibroblast, and islet β cell. HIF-1α signaling regulates cellular metabolism, triggering the release of inflammatory cytokines and inflammatory cells proliferation. It is known that microenvironment hypoxia, vascular proliferation, and impaired immunological balance are present in autoimmune diseases. To date, HIF-1α is recognized to be overexpressed in several inflammatory autoimmune diseases, such as systemic lupus erythematosus (SLE), rheumatoid arthritis, and function of HIF-1α is dysregulated in these diseases. In this review, we narrate the signaling pathway of HIF-1α and the possible immunopathological roles of HIF-1α in autoimmune diseases. The collected information will provide a theoretical basis for the familiarization and development of new clinical trials and treatment based on HIF-1α and inflammatory autoimmune disorders in the future.

## 1 Introduction

HIF-1α involves in metabolic pathway, which regulates immune cell function and inflammation (1, 2). The gene encoding HIF-1α is located at chromosome 14q21-q24 (3). Under a normal oxygen condition, proline hydroxylase (PHD) binds to HIF-1α and then combines with E3 ubiquitin ligase (VHL), triggering proline hydroxylation-ubiquitination and proteasomal degradation of HIF-1α (4). In the absence of oxygen, effects of the metabolic pathway is inhibited, and much HIF-1α accumulated in the nucleus, forming the active HIF-1α heterodimer with HIF-1β. Then, the activated HIF-1 binds to hypoxia response element (HRE) in the DNA and regulates expression of target genes related to angiogenesis, apoptosis, and cell migration (5). Expression of HIF-1α was up-regulated in response to tumor necrosis factor-α (TNF-α), interleukin-17A (IL-17A), phosphatidylinositol-3-kinase (PI3K) stimulation, which regulates the homeostasis of immune cells. HIF-1α also plays an anti-infection role in innate immune cells when they sense microorganisms (6). For example, after infecting with mycobacterium tuberculosis, HIF-1α in macrophages increase phagocytosis and accelerate glucose metabolism (7). Hypoxia alters the phenotype of dendritic cells, allowing naive T cells to differentiate into Th2 cells (8). To date, overexpression of HIF-1α was detected in the serum, skin tissue, and urine of different inflammatory autoimmune diseases, such as systemic lupus erythematosus (SLE) (9), rheumatoid arthritis (RA) (10), systemic sclerosis (SSc) (11), and psoriasis (12). In addition, functional studies *in vivo* and *in vitro* suggested an important role of HIF-1α in the pathogenesis of these diseases. Interestingly, targeting HIF-1α makes a potential for alleviating inflammatory disorders (13). Therefore, this review summarized the molecular mechanism of HIF-1α and discussed the function of HIF-1α in immune cells, particularly the relationship between HIF-1α and inflammatory autoimmune diseases.

## 2 HIF-1α signaling pathway

As a nuclear transcription factor, HIF-1α enters the nucleus after binding to HIF-1β, and then activates downstream signaling pathways, inducing generation of inflammatory components, vascularization and cell proliferation (14). Reactive oxygen species (ROS) reflects oxidative stress and cellular inflammatory metabolism, and expression of ROS is elevated under hypoxic condition. Substantial ROS stimulates NF-κB and inhibits activity of PHD and HIF asparaginyl hydroxylase (FIH), allowing HIF-1α to accumulate in the cytoplasm (15, 16). G protein-coupled receptor kinase 2 (GRK2)/HIF-1α are highly expressed after ROS stimulation and then affect expression of nucleotide-binding oligomerization structure-like receptor family Pyrin domain protein 3 (NLRP3) (17). NLRP3 contributes to pro-IL-1β and pro-IL-18 maturation with the aid of enzymes, which will activate IL-1β, IL-18 and lead to cytolytic death. Conversely, low levels of HIF-1α reduce expression of target genes, including phosphoinositide-dependent protein kinase-1 (Pdk1) and glucose transporter type 1 (Glut1), which in turn affect the glycolytic pathway and cellular ATP supply (18). Epidermal growth factor (EGF) and insulin-like growth factor-1 (IGF-1) activate PI3K. Activated PI3K then activates protein kinase B (Akt) on the cell membrane (19). In addition, mammalian target of rapamycin (mTOR) was activated by PI3K/Akt, which then increases HIF-1α expression. Elevated expression of HIF-1α up-regulates expression of VEGF, promoting vascular expansion (20). IL-23-induced glycolysis is diminished after inhibiting the Akt/mTOR/HIF-1α pathway (21). (AMP)-activated protein kinase (AMPK) is activated by SIRT3 stimulation, which then inhibits mTOR/HIF-1α pathway and induces less cell growth and more apoptosis (22–24).

IL-6 binds to gp160, then activates STAT3/HIF-1α, which promotes the proliferation of Foxp3^+^ regulatory T (Treg) cells and reduces activity and migration of hemangioma-derived stem cells (25, 26). IL-17 induces defective autophagy through interacting with STAT3/HIF-1α and causes inflammatory death of keloid fibroblasts (27). In addition, Janus kinase (JAK) signaling, an important upstream activator of STAT3, directly promotes NLRP3 expression and IL-1β secretion to aggravate inflammation (28). The classical mitogen-activated protein kinases (MAPK) and regulated extracellular protein kinases (ERK) pathways adapt to hypoxia, and then activate HIF-1α, thereby protecting cell development and avoiding oxidative damage (29, 30).

**Figure 1:**
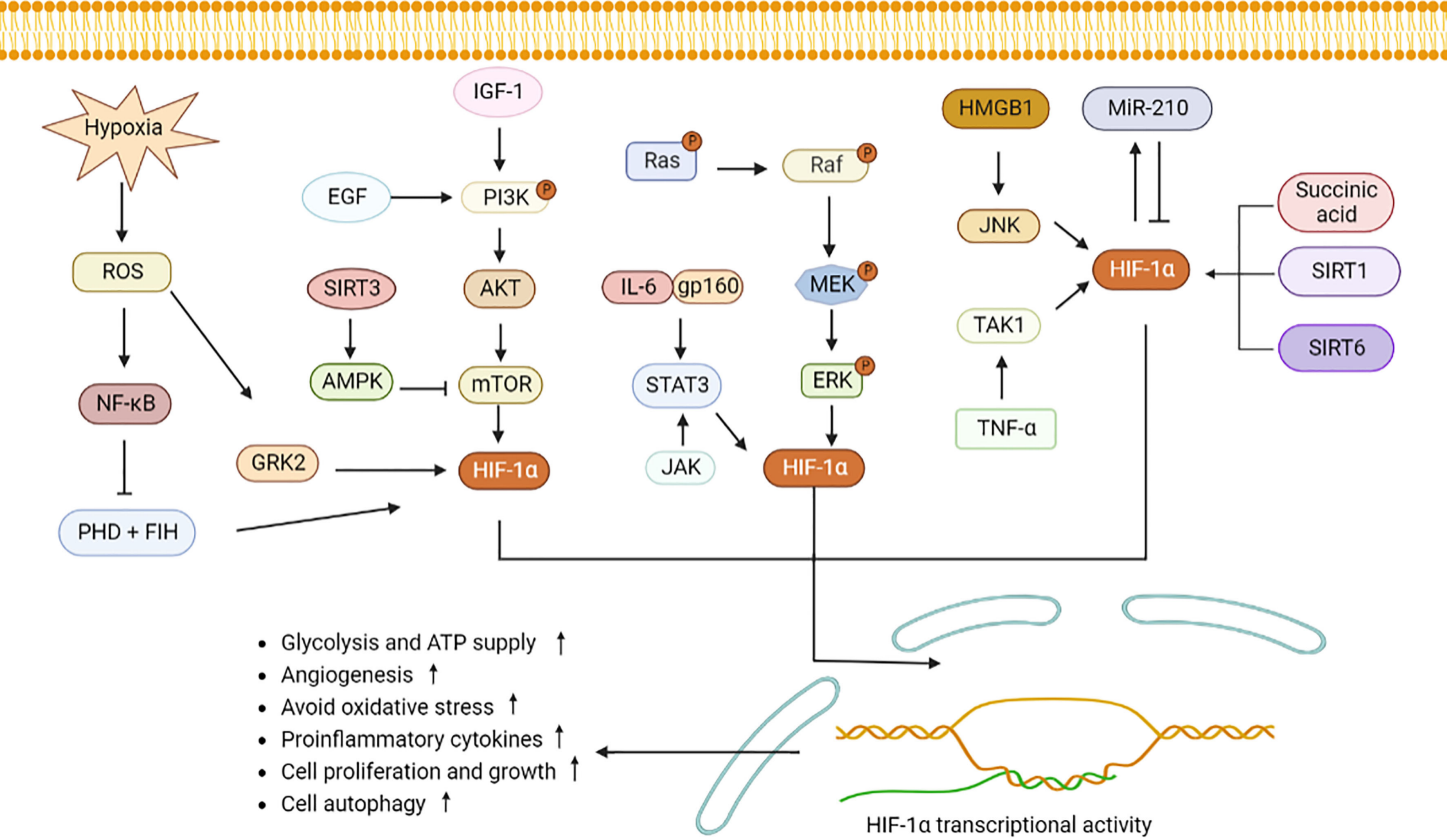
HIF-1α signaling pathway induces inflammatory response and metabolic changes. Hypoxia induces GRK2 and NF-κB expression by stimulating ROS and increases HIF-1α expression. PHD and FIH can be inhabited by NF-κB, leading to accumulation of HIF-1α. PI3K stimulated by EGF and IGF-1 induces Akt and mTOR to elevate HIF-1α secretion. SIRT3 activates AMPK to decrease mTOR expression. IL-6 binds to gp160, JAK, and ERK, and then stimulates STAT3/HIF-1α signaling. Similarly, succinic acid, SIRT1 and SIRT6 increase the intracellular expression of HIF-1α. MiR-210 interacts with HIF-1α. High level of HIF-1α promotes angiogenesis, cell migration and invasion, increases pro-inflammatory cells differentiation and cytokines production. ROS, reactive oxygen species; GRK2, G protein-coupled receptor kinase 2; PHD, proline hydroxylase; FIH, HIF asparaginyl hydroxylase; STAT3, signal transducer and activator of transcription 3; PI3K, phosphatidylinositol-3-kinase; Akt, protein kinase B; mTOR, mammalian target of rapamycin; IL-6, interleukin-6; NF-κB, nuclear factor-κB; JAK, Janus kinase; MAPK, mitogen-activated protein kinases; ERK, regulated extracellular protein kinases; HMGB1, high mobility group protein 1; JNK, c-Jun N-terminal kinase; TNF-ɑ, tumor necrosis factor-alpha; TAK1, transforming growth factor-activated kinase 1; miR-210, microRNA-210; AMPK, adenosine monophosphate activated protein kinase; SIRT1, sirtuin 1.

Under hypoxia, high mobility group proteins 1 (HMGB1) accelerated the c-Jun N-terminal kinase (JNK) pathway to stimulate HIF-1α/vascular endothelial growth factor (VEGF) axis, which is conducive to angiogenesis (17). In addition, TNF-α interacts with transforming growth factor-activated kinase 1 (TAK1), and promotes HIF-1α expression and cell glycolytic (31). MicroRNA-210 (miR-210), a marker of hypoxia, is regulated by HIF-1α. Since the 3’UTR of HIF-1α contains a non-canonical miR-210 target site, miR-210 also negatively inhibits HIF-1α expression by binding to this target (2, 32). Elevated miR-210 suppressed expression of HIF-1α target genes Glut1, p53 and fas, therefore protecting cell against hypoxia-induced apoptosis. Succinic acid, SIRT1/6 are two upstream signals for HIF-1α activation. After transporting from mitochondria to cytoplasm, succinic acid inhibits PHD activity, and activates HIF-α, leading to increased expression of IL-1β and inflammation (33). SIRT1 binds to the HIF-1α inhibitory domain (ID) and protects HIF-1α from deacetylation (34). Overexpression of SIRT6 inhibits the ubiquitination-protease system and favors HIF-1α accumulation, resulting in increased expression of VEGF, Ang1, Ang2, endothelin-1 (EF-1), and platelet-derived growth factor-BB (PDGF-BB), and promoting migration, invasion, and proliferation of human umbilical vein endothelial cells (35). When retinoic acid related orphan nuclear receptor γt (ROR γt) was subjected to HIF-1α, a trimer composed of P300, ROR γt, and HIF-1α fosters Th17 cells differentiation and IL-17 secretion (2). All these revealed that HIF-1α may involve in cytokines secretion and regulation of cellular function through downstream signaling pathways (**Figure 1**).

## 3 HIF-1α and immune cells

HIF-1α accumulates in nucleus due to hypoxia or external stimulation, which then induces proliferation, and metabolic changes in innate immune cells, such as macrophages, neutrophils, dendritic cells, natural killer cells, and mast cells. As for adaptive immunity, a few studies have focused on the relationship between T, B cells and HIF-1α. Nevertheless, available evidence showed that HIF-1α regulates inflammatory cytokines secretion, leading to imbalance of Th1, Th2, Th17, Treg cells, and CD8^+^ T cells that are participating in autoimmune disorders.

### 3.1 Role of HIF-1α in innate immune cells

#### 3.1.1 Macrophage

Macrophages have strong deformation movement and phagocytosis ability, and are involved in antigen presentation. When monocytes differentiate into macrophages, expression pattern of HIF-1α changes (36). Localization of HIF-1α shifts from the cytoplasm of monocytes to the nucleus of macrophages (36, 37). More monocytes were differentiated into macrophages under low oxygen condition, and there was higher expression of HIF-1α in differentiated macrophages (38). Therefore, the increase of glycolysis may be an inevitable result for monocytes-derived macrophages in a hypoxic microenvironment. Since then, increased HIF-1α will up-regulate function of macrophages, such as antigen presentation and inflammatory cytokines secretion (39). Elevated HIF-1α in macrophages also resists infection, which are able to kill and clear pathogens, such as mycobacterium tuberculosis, severe acute respiratory syndrome coronavirus 2 (SARS-CoV-2), and some fungi (7, 40–42). There are two types of macrophages, M1 and M2 macrophages. HIF-1α is activated in both cells (43, 44).

#### 3.1.2 Neutrophil

Neutrophils are one of the most abundant leukocytes in peripheral blood with chemotaxis and phagocytosis. HIF-1α regulates neutrophils survival under hypoxic condition, which depends on NF-kappa B (NF-κB) activation and ROS production (45, 46). Elevated HIF-1α causes neutrophils to exhibit antimicrobial activity. High level of HIF-1α in neutrophils before infection enhances reactive nitrogen species (RNS) production, which will lessen the mycobacterial burden (47). HIF-1α-deficient (HIF-1α^-/-^) mice are susceptible to bacterial infection and have negative response to vaccine (6). Activation of HIF-1α in zebrafish reduced apoptosis of neutrophils, delayed the improvement of inflammation, exhibiting pro-inflammatory properties (48). A dual host defense mechanism known as neutrophil traps (NETs) can resist germs, harm tissue, and blood vessels as a result of inflammation (49, 50). Blocking HIF-1α inhibits the extracellular bactericidal impact of NETs (50). Treatment of neutrophils with IL-4 inhibited HIF-1α-dependent hypoxic survival, which then limited production of pro-inflammatory components such as CCL2, CCL3, and TNF-α (51). Lipopolysaccharides (LPS) stimulation triggered lactate release by up-regulating glycolysis, NADPH-oxidase-mediated ROS and HIF-1α levels in bone marrow neutrophils (52). There was decreased glycolysis and lactate accumulation in bone marrow neutrophils from HIF-1α^-/-^ mice (52). Lactate induced mobilization of bone marrow neutrophils into peripheral blood and recruitment to the liver, leading to bone marrow neutropenia (52). Activating transcription factor 3 (ATF3) deficient (ATF3^-/-^) mice showed increased percentage of intrahepatic neutrophil trafficking, elevated expression of pro-inflammatory mediators IL-17A, CCL1, CCL2, and increased HIF-1α activity. Silencing of HIF-1α in ATF3^-/-^ mice inhibited neutrophil trafficking and production of IL-17A, CCL1, CCL2 in liver (53). In conclusion, HIF-1α is a global regulator of neutrophil inflammation and makes a role for anti-bacterial infection (54).

#### 3.1.3 Dendritic cell

Dendritic cells (DCs) are the most effective antigen-presenting cells and act as a bridge between innate and adaptive immunity (55). DCs in anoxic tissues showed high expression of HIF-1α (56). Hypoxia and LPS stimulation led to HIF-1α accumulation in DCs, along with reduced biological activity of proline hydroxylase (57, 58). HIF-1α alone, or interacts with target gene Glut1, glycolytic enzymes enhance glycolysis and ATP production (59). HIF-1α^-/-^ mice showed increased IL-22 secretion under hypoxia (56). Moreover, HIF-1α interacts with PI3K/Akt pathway to enhance migration ability of DCs (60). HIF-1α binding to p38 MAPK or long noncoding RNA Dpf3 (Lnc-Dpf3) will inhibit the reprogramming of glycolytic metabolism of DCs (61). HIF-1α^-/-^ immature DCs showed low expression of surface molecules MHC-II, CD80, CD86 (62). Coculturing HIF-1α^-/-^ immature DCs with CD4^+^ T cells or coculturing HIF-1α^-/-^ immature DCs with CD8^+^ T cells in the presence of LPS, TNF-α led to less proliferation of CD4^+^ T cells, CD8^+^ T cells (62). Similarly, HIF-1α^-/-^ mice had low titers of IgG antibody after vaccination, suggesting that HIF-1α deficiency may impair antigen presentation ability of DCs (62). Furthermore, maturation of DCs is negatively regulated by HIF-1α/NOS axis during mycobacterium tuberculosis infection (61). Silencing HIF-1α in DCs down-regulates the anti-fungal effect of DCs (63). Collectively, HIF-1α plays a role in antigen presentation, glycolysis reprogramming, and antimicrobial resistance of DCs.

#### 3.1.4 Natural killer cell

Natural killer (NK) cells mainly maintain anti-tumor and anti-infection effects in innate immune response (64). In human cardiomyocytes (HCMS), HIF-1α up-regulates expression of major histocompatibility complexes I-related molecule A/B (MICA/B), and then enhances the cytotoxicity of NK cells during hypoxia-reoxygenation (65). HIF-1α induces MICA expression to amplify the killing ability of NK cells. Loss of HIF-1α in NK cells leads to nonproductive angiogenesis to suppress tumors (66). HIF-1α^-/-^ NK cells fail to control cytomegalovirus viral load, resulting in increased morbidity (64). In addition, IL-15 activates STAT3 pathway and IL-2 activates PI3K/mTOR signaling, which then stabilize HIF-1α expression, and maintain natural defense against microbial infection and tumor development (67, 68). Thus, HIF-1α plays a role in regulating NK cell glucose metabolism, anti-tumor, and anti-infection during hypoxia.

#### 3.1.5 Mast cell

**Figure 2:**
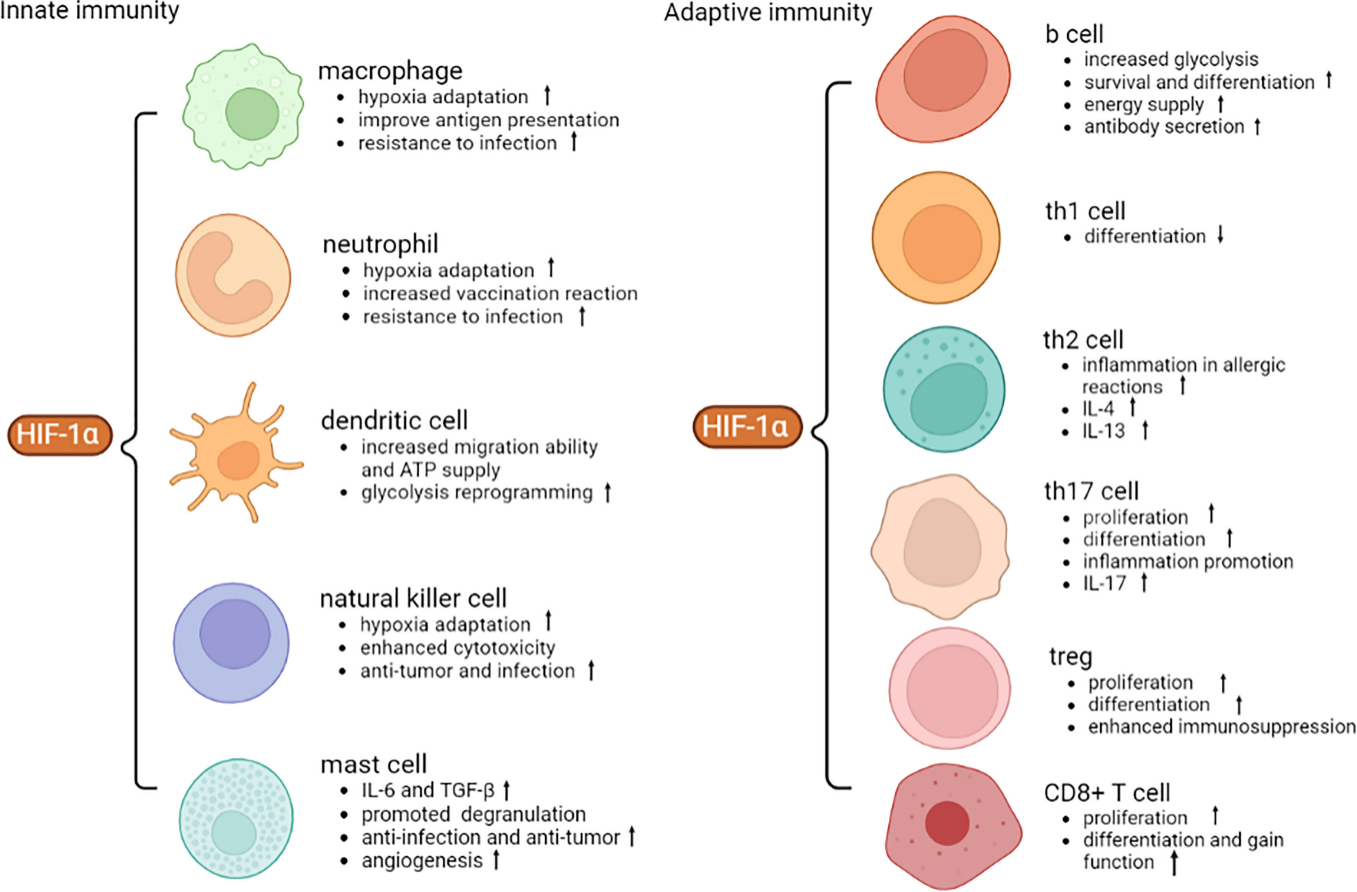
Role of HIF-1α in different immune cells. HIF-1α facilitates anti-infection, anti-tumor and hypoxic adaptation of innate immunity. HIF-1α promotes the proliferation and differentiation of adaptive immune cells and secretion of inflammatory cytokines. VEGF, vascular endothelial growth factor; ATP, adenosine triphosphate; IL-4, interleukin-4; TGF-β, transforming growth factor-β.

Mast cells are involved in inflammation and type I hypersensitivity. HIF-1α is expressed in mast cells of human and animal melanoma tissues (69). In LAD2 mast cells, HIF-1α knockdown attenuates IL-6 release after Toll-like receptor 4 (TLR4) stimulation (70). Similarly, silencing HIF-1α reduces mast cells degranulation and down-regulates expression of TGF-β, and VEGF (71). In ovalbumin (OVA) vaccination-treated mice, administration of HIF-1α increases vascular permeability and plasma exudation through the PI3K/VEGF signaling axis (6). Desferrioxamine treatment leads to elevated expression of HIF-1α in human mast cell 1 (HMC-1), and promotes IL-6, IL-8, TNF-α production in mast cells by activating HIF-1α or NF-κB signaling (72). Lactic acid interrupts miR-155-activated HIF-1α, leading to diminished IL-33 secretion in mast cells (73). Treatment of melanoma mice with H1-receptor antagonist blocks HIF-1α expression and suppresses tumor growth and mast cells infiltration, suggesting that mast cell-derived HIF-1α accelerates melanoma growth (74). MC extracellular traps (MCETs) are formed as a result of phagocytosis of MCs, which produce antimicrobial peptides (74). Enhancement of HIF-1α activity leads to elevated anti-bacterial activity of MCs by inducing MCETs. Conversely, mice lacking HIF-1α are more susceptible to bacterial infection (75) (**Figure 2**).

### 3.2 Role of HIF-1α in adaptive immune cells

#### 3.2.1 B cell

HIF-1α involves the glycolytic process of B cells and affects their differentiation, maturation, antibody secretion and viability. Following LPS and IL-4 stimulation, HIF-1α^-/-^ germinal center (GC) B cells have reduced expression of glycolytic genes and glycolytic rate-limiting enzymes, including GAPDH, M2-type pyruvate kinase (PKM2) (76). Similarly, loss of von Hippel-Lindau tumor suppressor protein (VHL) in B cells allows excessive stabilization of HIF-1α in B cells, thereby interfering with the balance of glycolysis and aerobic metabolism (77). HIF-1α^-/-^ B220^+^ bone marrow cells have lower glycolytic capacity than wild-type cells. This process is due to restricted expression of genes encoding glucose transporters, including phosphofructokinase 2 and fructose-2,6-bisphosphate kinase (78). HIF-1α-regulated glycolysis is important for early pre-B cells and IgM^+^ B cells, however, blocking glycolysis using 2-DOG does not slow down pre-B cells differentiation into immature B cells, suggesting that HIF-1α is required for different stages of B cells (78). Similarly, HIF-1α activity is higher in bone marrow pro-B cells and pre-B cells, and is lower in immature B cells (79). HIF-1α limits pyruvate entry into tricarboxylic acid cycle (TCA), and B cells with HIF-1α deficiency can transport more pyruvate and generate energy in the respiratory chain (78). In addition, binding of HIF-1α to HRE of IL-10 gene promoter increases IL-10 secretion in B cells (80), and regulates innate-like B cells and B10 differentiation, resulting in decreased IgM secretion (81). Splenic B cells from HIF-α^-/-^ mice were cultured with hypoxia condition, showing increased expression of IL-10 in B cells as compared to that in normoxia (80). When naive CD4^+^ T cells were co-cultured with CD1d^hi^CD5^+^ B cells from HIF-ɑ^-/-^ mice, there were high percentage of CD4^+^IFN-γ^+^, CD4^+^IL-17A^+^ T cells, and increased expression of IFN-γ, IL-17A (80). Overexpression of HIF-1α in RA synovial fibroblasts (RASFs) promoted expression of IL-6, IL-8, TNF-α, and IL-1β (82), and co-culturing HIF-1α^-/-^ RASFs with allogenic CD19^+^ B cells down-regulated expression of stromal cell-derived factor (SDF)-1, vascular cell adhesion molecule (VCAM)-1, IgG and up-regulated percentage of CD19^+^CD24^hi^CD27^+^ B10 cells, CD19^+^CD27^+^IgD^+^ innate-like B cells, expression of natural IgM (82). ROS activates tyrosine kinase and promotes nuclear factor (erythroid-derived 2) like 2 (Nrf2), HIF-1α to improve B cells survival (83). In Wil2-NS B cells under hypoxia, Nrf2 and HIF-1α promote expression of C-X-C chemokine receptor type 4 (CXCR4) and increase viability of B cells (84). HIF-1α was highly expressed in GC B cells. Knockout HIF-1α in B cells impaired GC reaction, leading to defective class-switch recombination and production of high-affinity plasma cells (76).

#### 3.2.2 Th1 cell

Coculturing HIF-1α^-/-^ antigen-presenting cells (APCs) with Th1 cells does not induce Th1 cells expansion (85). HIF-1α selectively induced secretion of IL-12p40 to interrupt differentiation of naive T helper cells into Th1 cells, limiting mucosal inflammation (86). Under hypoxia, increased phosphorylation of STAT3 in Th1 cells contributes to transcription of HIF-1α, which in reversely inhibits transcription of cell signal transduction inhibitor 3. Consequently, this positive feedback enhances STAT3 activation and down-regulates Th1 response. Furthermore, Th1 cells under hypoxia lost the ability to secrete IFN-γ. HIF-1α limits Th1 cells differentiation through inhibiting production of IL-12 under hypoxia (87, 88). In addition, treatment with miR-182, an inhibitor of HIF-1α, accelerates Th1 cells differentiation (89).

#### 3.2.3 Th2 cell

Th2 cells involve anti-infection, asthma, and other hypersensitivity responses. More Th2 cells differentiation and increased VEGF expression were observed in OVA-induced asthma mice model, and there was high expression of HIF-1α in the mice lung tissue (90). In HIF-1α^-/-^ mice exposed to cobalt, expression of IgE, leukotriene C4 (LTC4), eosinophil cationic protein (ECP) was decreased in alveolar lavage fluid and lung tissue (91). In mice with HIF-1α^-/-^ DCs, secretion of Th2 cytokines, such as IL-5, IL-10, and IL-13 was reduced (91). Under hypoxic condition, expression of membrane binding protein CD44 on DCs is increased, which then promotes Th2 cells polarization, accompanied by increased IL-4 secretion (8). Usage of anthraquinone, a HIF-1α inhibitor, is able to restrain HIF-1α expression, and inhibits differentiation of Th2 cells and expression of IL-4, IL-13 (92–94). During infection with pathogens, HIF-1α expression is increased in Th2 cells, leading to Th2 cells proliferation (95).

#### 3.2.4 Th17 cell

Evidence suggests that HIF-1α is a key molecule regulates activities of Th17 cells and expression of IL-17 (1, 96). It is known that RORγt is the transcription factor for Th17 cell. HIF-1α deficiency inhibits Th0 cells developing into Th17 cells and down-regulates RORγt expression (97). Escherichia coli infection increases the amount of HIF-1α in the liver, which then induces Th17 cells differentiation by increasing IL-6 expression (98). At condition of 5% O_2_, HIF-1α is activated (99), and there are elevated percentages of Th17 cells and expression of IL-6 (100). Treatment with metformin and epigallocatechin-3-gallate (EFCG) inhibits the mTOR signaling, thereby inhibiting HIF-1α expression and Th17 cells differentiation (101, 102). In Adipor1^-/-^CD4^+^ T cells, there was reduced glycolysis metabolism and Th17 cells polarization, which is due to disturbance of HIF-1α (103). In addition, HIF-1α is a target gene of miR-210. MiR-210 directly reduces the transcription of HIF-1α to delay differentiation of Th17 cells (32).

#### 3.2.5 Regulatory T cell

Treg cells, including natural regulatory T cells and inducible regulatory T cells, are a class of cells with inhibitory effects in immune response. During different Th cells metabolism and differentiation, Th1 and Th17 utilize high levels of glycolytic metabolism to provide capacity for their proliferation, whereas Treg cells require aerobic metabolism to enhance their inhibitory function (104). HIF-1α promotes CD73 expression in Treg cells and binds to CD73 to expand Treg cells to convert ATP into immunosuppressive adenosine (105). Increased expression of O_2_ at the tumor site down-regulates HIF-1α to affect tumor cell metabolism and negatively regulates Treg cells differentiation (106). Under hypoxia, transfection of CD4^+^CD25^+^ T cells with lentiviral vector containing low expression of HIF-1α increases expression of Foxp3, which induces Treg cells differentiation and immunosuppressive function (107). IL-1β up-regulates HIF-1α expression to inhibit Treg cells polarization in response to inflammatory stimuli (108).

#### 3.2.6 CD8^+^ T cell

As HIF-1α^-/-^CD8^+^ T cells were differentiated into effector cytotoxic T lymphocytes (CTLs), there was reduced expression of genes regulating glycolytic metabolism, such as *Hk2*, *Pdk1*, *Mct4* and *PgK*, and less glucose uptake and lactate production (109). HIF-1α^-/-^ effector CD8^+^ T cells did not down-regulate surface expression of CD62L, but down-regulated expression of IFN-γ, TNF-α. Hypoxia increased expression of the cytolytic molecule granzyme B, activation-related costimulatory molecules CD137, OX40, GITR, and checkpoint receptors PD-1, TIM3, VEGF-A and LAG3 (109), which was obtained in HIF-1α^-/-^CD8^+^ T cells in response to IL-2 stimulation as well. HIF-1α^-/-^ effector CD8^+^ T cells showed a reduced ability to kill target cells (109). Deficiency in NIX-dependent mitophagy results in metabolic defects in effector memory CD8^+^ T cells, and NIX deficiency promoted HIF-1α accumulation, altering cellular metabolism from long-chain fatty acid to short/branched-chain fatty acid oxidation, thereby compromising ATP synthesis (110). Inhibiting HIF-1α accumulation restored long-chain fatty acid metabolism and effector memory CD8^+^ T cells formation, suggesting that HIF-1α regulates effector memory CD8^+^ T cells formation by NIX-mediated mitophagy (110). High activity of HIF-1α in tumor microenvironment down-regulated infiltration and activity of CD8^+^ T cells (111). There was elevated T cells infiltration at early stage of tumorigenesis in the tumor site along with up-regulated percentage of memory CD4^+^, CD8^+^ T cells (112). Inhibition of HIF-1α down-regulated expression of pro-inflammatory factors IL-10, IL-12, PGE2, S-180, TNF-α, and abrogated memory CD4^+^, CD8^+^ T cells-mediated suppression of tumor-associated macrophages (TAM) (112). Knocking down HIF-1α negative regulator von Hippel-Lindau (VHL) in CD8^+^ T cells led to differentiation of tissue-resident memory-like (Trm-like) tumor-infiltrating lymphocyte (TIL), by which VHL^-/-^ TILs accumulated in tumors and showed a core Trm signature, indicating that HIF-1α activity in CD8^+^ TILs up-regulates accumulation and antitumor activity (113). Similarly, VHL^-/-^CD8^+^ effector T cells did not express KLRG1, a marker of T cell terminal differentiation, suggesting a positive effect of HIF-1α on CD8^+^ T cells differentiation (114).

## 4 HIF-1α and autoimmune diseases

### 4.1 Systemic lupus erythematosus

SLE is a typical inflammatory autoimmune disease characterized by production of autoantibodies and damage to multiple tissues and organs, such as skin, joints, and kidneys. Lupus nephritis (LN) is the mostly complicated disease in SLE, which is also the major cause of incidence and mortality in lupus patients (115).

Urinary HIF-1α levels are higher in LN patients compared with that in healthy controls, and were associated with histologic chronicity indexes and the estimated glomerular filtration rate (eGFR) in LN patients (116). In LN patients and MRL/lpr lupus mice, expression of HIF-1α in both glomerular and tubulointerstitial areas was increased and percentage of intraglomerular HIF-1α^+^ cells was increased (9). The levels of intraglomerular HIF-1α were related to renal pathology activity index and clinical manifestations in LN patients. In SLE patients’ CD4^+^ T cells, HIF-1α was overexpressed (2). Regarding gene single-nucleotide polymorphism (SNP) and SLE risk, a study showed that there are no significant differences in genotypes frequencies between the patients with SLE and the controls (rs11549465, rs12434438, rs1957757, rs1951795, rs7143164) (117). Silencing HIF-1α in MRL/lpr mice can inhibit serum levels of IL-17, anti-nucleosome antibody, proteinuria, IgG and C3 depositions in kidney (1). Inhibition of glutaminase in MRL/lpr mice affects the glycolysis pathway by reducing HIF-1α expression and decreases percentage of CD3^+^CD4^-^CD8^-^ T cells, urine albumin, and glomerular renal pathology scores (118). Thus, HIF-1α may be a promising target for treatment of lupus.

### 4.2 Rheumatoid arthritis

RA is a chronic disease with symmetry arthritis as its main clinical manifestation, which is characterized by synovial hyperplasia and osteoarticular destruction (119). HIF-1α expression was increased in serum, sublining layer in synovial membrane from RA patients (10, 120, 121). The number of HIF-1α^+^ cells in RA synovial tissue is correlated with blood vessels, inflammatory endothelial cells infiltration, proliferation, and synovial score (119). Moreover, expression of HIF-1α was reinforced in collagen-induced arthritis (CIA) mice (122–125).

In CIA mice treated with hyperbaric oxygen, there was elevated percentage of Treg cells accompanied by lower expression of HIF-1α. Pannus formation represents a distinctive pathological feature of RA, and VEGF mediates arthropathic proliferative angiogenesis in arthritis. In adjuvant-induced arthritis (AA) rats and RA patients, expression of HIF-1α was positively related to expression of VEGF, and increased HIF-1α accelerated synovial angiogenesis and resulted in joint inflammation (126, 127). On the contrary, inhibition of HIF-1α expression in AA rats showed opposite effects (128). It is known that erosion and destruction of articular cartilage is a prominent pathological feature of RA. Under hypoxic condition, fibroblast-like synovial cells in RA (RA-FLSs) transformed into epithelial mesenchyme, and HIF-1α promoted migration and invasion of the cells *via* STAT3/HIF-1α/fascin-1 axis (129, 130). NF-κB interacts with HIF-1α to promote the enzymatic activity of matrix metalloproteinases 2 (MMP2) and MMP9, and then disrupts histological barrier and destroys bone material (131). When CD14^+^ monocytes differentiate into osteoclasts, there was elevated expression of HIF-1α in osteoclasts (132). HIF-1α increases osteoclasts-mediated bone resorption (133). In RASFs, HIF-1α overexpression induces Th1 and Th17 cells expansion and increases expression of INF-γ and IL-17 (82, 134). HIF-1α inhibitor, Pyridine formamide compound AMSP-30m, facilitated synovial cells apoptosis (125). Citrullinated proteins are considered as a biomarker of RA. Knocking out HIF-1α in RASFs decreased citrulline protein (135). CIA mice treated with IL-34, succinate, and sinomenine up-regulated Ang-1 expression *via* the HIF-1α/VEGF axis (128, 136, 137). IL-38, an inflammatory related cytokine, exerts angiopoietin-inhibiting and anti-inflammatory function in CIA mice (138). Activation of PI3K/Akt/HIF-1α and NK-κB/HIF-1α signaling pathways augmented migration and invasion of RA-FLSs (129, 131). HIF-1α is capable of up-regulating osteoclasts-mediated bone resorption (139), whereas IL-38 contributed to secretion of osteogenic factors through SIRT1/HIF-1α signallings (129). Therefore, expression of HIF-1α was increased in arthritis and may promote arthritis development by downstream signals.

### 4.3 Inflammatory bowel disease

Inflammatory bowel disease (IBD), including ulcerative colitis (UC) and Crohn’s disease (CD), are a class of chronic intestinal inflammatory diseases characterized by intestinal barrier dysfunction and intestinal mucosal hypoxia. Compared with controls, higher expression of HIF-1α exists in intestinal cells and M1-type macrophages of CD patients (140, 141). In terms of population susceptibility, HIF-1α gene rs11549467 polymorphism did not correlate with IBD risk in Moroccan population (142).

The HIF-1α/glycolytic pathway disrupts balance of M1/M2 macrophages and the secretion function of neutrophils to affect the pathological state of colitis (143–145). Succinate is an intermediate product of the tricarboxylic acid cycle that drives HIF-1α to stimulate IL-1β production and aerobic glycolysis in M1 macrophages, favoring the M1 phenotype (146). M2 macrophages, on the other hand, acquire energy mainly from fatty acid metabolism and oxidative metabolism (146). Tiliroside attenuates disease activity in mice with colitis, where it promotes HIF-1α enzyme degradation (143). In clinical trials with cyclosporine from UC patients, cyclosporine increased HIF-1α expression and glycolysis in neutrophils, accompanied by release of antimicrobial peptides, ROS, and myeloperoxidase (MPO) (145). CD-associated Escherichia coli activated VEFG in intestinal epithelial cells, triggering angiogenesis (147). HIF-1α interacted with IL-33 at the promoter region and is able to stabilize IL-33-induced mucosal homeostasis (148). Inhibition of PHD1 stabilizes HIF-1α levels, and then protects the intestinal mucosa (149). Furthermore, treatment of Bifidobacterium IL-10 inhibited inflammation in colitis mice by restoring Treg/Th17 balance (150). Dimethyloxalylglycine (DMOG) is a hydroxylase inhibitor that stabilizes HIF-1α, and DMOG improved chronic intestinal inflammation (151). However, a study revealed that mice with HIF-1α deficiency in DCs lost much weight and exhibited severe intestinal inflammation after dextran sodium sulfate (DSS) treatment. HIF-1α plays a protective role in DCs (152), T cells (153), and epithelial cells (154) in murine colitis. Inhibition of HIF-1ɑ in myeloid cells exacerbated infiltration of neutrophils and Ly6^+^ monocytes in lesion tissues, and HIF-1α^-/-^ colonic macrophages had a reduced pro-resolving profile (155). Therefore, HIF-1α signaling contributes to colitis resolution.

### 4.4 Systemic sclerosis

Systemic sclerosis (SSc) is an autoimmune disease featured by autoimmunity, vascular lesions and interstitial fibrosis. Chronic hypoxia is a prominent feature in SSc, which can lead to vasculopathy and tissue fibrosis (11). It has been shown that expression of HIF-1α in human microvascular endothelial cell line-1 (HMEC-1) was up-regulated under hypoxia (11), and the skin tissue had much HIF-1α^+^ cells in patients with SSc (156). According to a study in French Caucasian population, HIF-1α gene polymorphism was associated with SSc risk. The frequencies of genotypes AG, GG in rs12434438 were higher in SSc patients than in controls (157). Another study in Japanese SSc patients obtained that AA genotype in rs12434438 was associated with SSc patients with severe pulmonary arterial hypertension (PAH), suggesting that rs12434438 polymorphism may relate to occurrence of SSc combined with PAH (158).

HIF-1α expression was closely related to VEGF expression in SSc patients (11). HIF-1α/VEGF axis induced vascular endothelial transformation into interstitial under hypoxia, leading to tissue fibrosis and vasculopathy (159, 160). Expression of connective tissue growth factor (CTGF) and HIF-1α was both rised in the skin of SSc patients, by which HIF-1α facilitated CTGF expression, and then resulted in skin fibrosis (161, 162). On the contrary, treatment with 2-methylestradiol diminished HIF-1α expression, reduced collagen synthesis, fibrocyte proliferation in fibroblasts, suggesting that targeting HIF-1α may give potential for treatment of SSc (161).

### 4.5 Psoriasis

Psoriasis is a chronic inflammatory disease characterized by excessive angiogenesis, proliferation of keratin-forming cells (163). Expression of HIF-1α was increased in both skin lesions, and serum from patients with psoriasis as compared to those in controls (12, 164–167). Ang-1, Ang-2, and Tie-2 are overexpressed in the papillary dermis of psoriatic skin, which are induced by HIF-1α. Expression of insulin-like growth factor-II (IGF-II) and VEGF in human keratinocytes cells (HaCat cells) was regulated by HIF-1α. Expression of HIF-1α positively correlated with microvessel density (164). MiR-150 restrains HaCat cells proliferation by binding to promoter of HIF-1α (168). It is accepted that increased proliferation and reduced differentiation of keratinocytes are characteristics of psoriasis. Stimulation of the cells with bone morphogenic protein 6 (BMP6) inhibited proliferation and promoted differentiation of keratinocytes. HIF-1α inhibited expression of BMP6 by binding to the HRE of promoter of BMP6, thereby aggravating the pathological features in psoriasis (168). Furthermore, HIF-1α bound to miR-210, suppressed expression of target genes *STAT6* and *LYN*, leading to Th17 cells differentiation in psoriasis mice (169).

### 4.6 Multiple sclerosis

Multiple sclerosis (MS) is a central nervous system disease caused by autoimmune inflammation, accompanied by demyelination, blood-brain barrier damage. Experimental autoimmune encephalomyelitis (EAE) mouse model is the classic animal model of MS.

In MS patients, white matters had hypoxia and high expression of HIF-1α (170). Similarly, EAE mice had elevated HIF-1α expression in mice tissues, and was related to the neurological defect (171). A case-control study discussed association between MS and HIF-1α polymorphism, showing no association of HIF-1α polymorphism and MS risk (172). In EAE mice, inhibiting HIF-1α expression leads to reduced intermittent hypoxia and promotes Treg cells differentiation and IL-10, TGF-β production (101). Treatment of MS patients with fumarate caused accumulation of HIF-1α, lowered the risk of MS recurrence (171). IL-1β induced expression of HIF-1α in astrocytes, changing the permeability of the blood-brain barrier in brain (173).

### 4.7 Type 1 diabetes mellitus

Type 1 diabetes mellitus (T1DM) is characterized by hyperglycemia, in which islet β-cell damage is mainly caused by autoimmunity. High expression of HIF-1α attenuated β-cell death and β-cell loss in islet (174). Induction of hypoxia in islet β-cell with CoCl (cobalt chloride) improved β-cell survival and relieved proteinuria and tubulointerstitial damage in diabetic rats, mediated by increased transcription of HIF-1α (175, 176). Thus, HIF-1α may protect against the hypoxia stress. Inhibiting expression of HIF-1α increased the infectivity of β cells to viruses, especially coxsackie viruses (177). Peripheral nerve damage, diabetic heart disease and diabetic nephropathy are some severe complications of diabetes. HIF-1α protects against peripheral nerves damage caused by hyperglycemia *via* inhibiting ROS, VEGF expression (178). P53 reduces cardiomyocyte apoptosis by increasing HIF-1α stabilization and ameliorating defects in glycolysis and angiogenesis. Similarly, a carbohydrate restriction diet (CR) can up-regulate HIF-1α expression and improve nephropathy in T1DM rats (179). Therefore, HIF-1α may suppress T1DM pathogenesis (**Table 1**).

**Table 1:** Expression of HIF-1ɑ in inflammatory autoimmune diseases.

Diseases	Sample	Expression	References
SLE	Urine	Increasea	116
	Glomerulus and Tubular	Increasea,b	9
	CD4+T cell	Increasea	2
RA	Synovial tissue	Increasea	10, 120, 121
	Serum	Increaseb	123–126
IBD	Intestinal cells	Increasea	140
SSc	Skin tissue	Increasea	162
Psoriasis	Skin lesion	Increasea	12, 164–166
	Serum	Increasea	167
MS	White matter	Increasea	170
	Tissue	Increaseb	171
T1DM	Islet tissue	Increasea	175

SLE, systemic lupus erythematosus; RA, rheumatoid arthritis; IBD, inflammatory bowel disease; SSc, systemic sclerosis; MS, multiple sclerosis; T1DM, type 1 diabetes mellitus.

a Human.

b Mice.

## 5 Conclusion

HIF-1α regulates angiogenesis and secretion of inflammatory cytokines by adapting to a hypoxic environment. In recent years, growing evidence has indicated that HIF-1α worked in several inflammatory autoimmune diseases. Functional studies suggest the effects of HIF-1α in the pathology of these disorders. For example, HIF-1α mediates excessive activation of innate immunity, leading to dysregulated biological function of innate immune cells, such as antigen presentation and anti-infection. Similarly, HIF-1α impacts cell proliferation and differentiation, pro-inflammatory cytokines release in adaptive immunity. However, some points need to be clarified in the future. Firstly, limited studies discussed polymorphisms in the HIF-1α gene and lupus, IBD. Gene polymorphism studies may provide basic data for the treatment and prevention of autoimmune disorders by revealing the risk of HIF-1α in autoimmune diseases, disease phenotype, and responsiveness to drug treatment. Thus, the above disorders require more studies with large samples and multiple races. Secondly, since HIF-1α is closely related to cell metabolism and energy supply, the relationship between HIF-1α and non-immune cells involved in the process of autoimmune diseases should be paid attention like cancer cells (180), renal tubular epithelial cells (9), and synovial cells (121). Thirdly, When Treg cells were subjected to hypoxia, high levels of HIF-1α stimulated proliferation of Treg cells and promoted the immunosuppressive effect. For instance, activation of the Akt/mTORC1 signaling pathway and subsequent activation of HIF-1α induces glucose transporter and glycolytic enzyme expression. HIF-1α increases the levels of pyruvate dehydrogenase kinase (PDK) and lactate dehydrogenase (LDH), inhibits the conversion of pyruvate to acetyl-CoA and promotes lactate production. The metabolic shift of Treg cells to aerobic glycolysis facilitates immunosuppressive function (181). However, in the presence of high levels of mTOR stimulator, Treg cells prefer aerobic glycolytic reprogramming accompanied by elevation of HIF-1α expression, thereby inhibiting Treg cells’ function. For Treg cells, the same pathway that inhibits their development may be necessary in functionally mature Treg cells (182). Therefore, when exploring the mechanism of HIF-1α in regulating Treg cells, different proliferation and differentiation stages, different metabolic patterns of Treg cells, and expression of mTOR signaling should be considered. Fourthly, in T1DM, HIF-1α protects pancreatic islet β-cell, and reduces the complications related to T1DM. Overexpressed HIF-1α protects against intestinal inflammation, and low expression of HIF-1α aggravates IBD. Interestingly, inhibition of HIF-1α expression in bone marrow cells and myeloid cells exacerbates intestinal inflammation, which contradicts its function in other diseases. Thus, the clear molecular mechanism for HIF-1α in different inflammatory autoimmune diseases needs specific discussion in the future.

Although some of the above limitations remain to be discussed to date, it is undeniable that HIF-1α performs significantly in inflammatory autoimmune diseases. This review can provide a theoretical basis for the development and application of HIF-1α as a disease marker and targeted drugs in the future.

## Author contributions

Study conception and design: Y-YT, W-DX. Acquisition of data, analysis and interpretation of data: D-CW, Y-QW, A-FH. Drafting the article: Y-YT, W-DX. Final approval of the version of the article to be published: all authors, and that all authors agree to be accountable for all aspects of the work. All authors contributed to the article and approved the submitted version.
